# Downregulation of phosphoglycerate mutase 5 improves microglial inflammasome activation after traumatic brain injury

**DOI:** 10.1038/s41420-021-00686-8

**Published:** 2021-10-12

**Authors:** Yuhua Chen, Kai Gong, Limin Guo, Bingchang Zhang, Sifang Chen, Zhangyu Li, Xu Quanhua, Wei Liu, Zhanxiang Wang

**Affiliations:** 1 Department of Central Laboratory, Xi’an Peihua University, Xi’an, 710125 China; 2 Trauma Center, First Affiliated Hospital of Xiamen University, 55 Zhenhai Rd, Xiamen, 361003 Fujian China; 3 Department of Anatomy and Neurobiology, Central South University, 172 Tongzipo Rd, Changsha, 410013 Hunan China; 4 Department of Neurosurgery, Xiamen Key Laboratory of Brain Center, The First Affiliated Hospital of Xiamen University, 55 Zhenhai Rd, Xiamen, 361003 Fujian China; 5 Department of Neurosurgery, Bijie Traditional Chinese Medical Hospital, Bijie, 551700 China

**Keywords:** Microglia, Trauma

## Abstract

Traumatic brain injury (TBI) is considered as the most common cause of disability and death, and therefore an effective intervention of cascade pathology of secondary brain injury promptly can be a potential therapeutic direction for TBI prognosis. Further study of the physiological mechanism of TBI is urgent and important. Phosphoglycerate mutase 5 (Pgam5), a mitochondrial protein, mediate mitochondrial homeostasis, cellular senescence, and necroptosis. This study evaluated the effects of Pgam5 on neurological deficits and neuroinflammation of controlled cortical impact-induced TBI mouse model in vivo and LPS + ATP-induced microglia model in vitro. Pgam5 was overexpressed post-TBI. Pgam5 depletion reduced pyroptosis-related molecules and improved microglia activation, neuron damage, tissue lesion, and neurological dysfunctions in TBI mice. RNA-seq analysis and molecular biology experiments demonstrated that Pgam5 might regulate inflammatory responses by affecting the post-translational modification and protein expression of related genes, including Nlrp3, caspase1, Gsdmd, and Il-1β. In microglia, Pgam5-sh abrogated LPS + ATP-induced Il-1β secretion through Asc oligomerization-mediated caspase-1 activation, which was independent of Rip3. The data demonstrate the critical role Pgam5 plays in nerve injury in the progression of TBI, which regulates Asc polymerization and subsequently caspase1 activation, and thus reveals a fundamental mechanism linking microglial inflammasome activation to Asc/caspase1-generated Il-1β-mediated neuroinflammation. Thus, our data indicate Pgam5 worsens physiological and neurological outcomes post-TBI, which may be a potential therapeutic target to improve neuroinflammation after TBI.

## Background

Traumatic brain injury (TBI), a global health challenge, is known to be one of the most common causes of death and disability in young men, and it imposes a burden on patients and their families, as well as a society [1, 2]. Typically, a complex set of pathophysiological processes leads to severe secondary brain injury within hours or days of acute brain injury, which follows the primary injury-mediated neuroinflammation, immune response, oxidative stress, mitochondrial dysfunction, and apoptosis [3, 4]. Neuroinflammation is believed to play an important role in secondary changes [4, 5]. Because in primary brain injury, the opportunity of treatment can be lost within a very short period, the effective intervention of cascade pathology of secondary brain injury promptly will become a potential therapeutic direction for TBI [6]. Thus, it is urgent and important to further study the physiological mechanism of neuroinflammation and nerve injury to alleviate the poor prognosis of TBI patients.

Phosphoglycerate mutase 5 (Pgam5), a mitochondrial protein, localized to the mitochondrial outer–inner membrane contact sites, its activation leads to a linear fracture of string-arranged mitochondria, and this phenomenon mediated mitochondrial and cellular fate [7], including cellular senescence [8] and early stages of necroptosis [9–11]. Some studies have suggested that Pgam5 drives necroptosis through imposing mitochondrial quality control via Drp1 phosphorylation [12, 13]. Pgam5 plays an important role in cardiac microvascular and skin ischemia-reperfusion injury through the Pgam5/CypD/mPTP pathway and Pgam5/Drp1 necrotic pathway [14, 15]. Previously, we demonstrated Pgam5 involved in the progression of neuronal injury following TBI via Drp1 activation-mediated mitochondrial dysfunction [16]. Furthermore, Pgam5 involves regulating Il-1β secretion in mouse bone marrow-derived dendritic cells (BMDCs) [10, 17]. However, whether Pgam5 is involved in Il-1β secretion and neuroinflammation post-TBI and by what pathway is still unclear.

Pyroptosis, a kind of inflammatory cell necrosis, is mainly characterized by the activation of caspase1 and the secretion of mature Il-1β and Il-18 and where Nlrp3 inflammasome acts as the pivotal regulatory signal of pyroptosis, which serves as a potential biomarker and therapeutic target [18]. In our previous studies, we found TBI was accompanied by increased neuroinflammation and pyroptosis during the acute phase post-TBI [19], which were significantly relieved by suppressing Nlrp3 inflammasome-mediated pyroptosis [19–21]. Thus, exploring the regulation mechanism of the NLRP3 inflammasome may provide a new idea for regulating the secretion of inflammatory factor Il-1β and neuroinflammation after TBI.

This study aims to explore the potential role and molecular mechanism of Pgam5 in the pathogenesis of TBI. Pgam5 depletion was used in vivo controlled cortical impact (CCI) TBI mouse model and in vitro lipopolysaccharide (LPS)-induced microglia models to demonstrate the role of Pgam5 in downstream neuroinflammation post-TBI. Our data indicate that Pgam5 facilitates physiological processes of TBI, which may be presented as a promising therapeutic target post-TBI.

## Materials and methods

### CCI model

We carried out our experiments on Pgam5-deficient (Pgam5^−/−^; Cyagen Biosciences Inc., Guangzhou, China; C57BL/6 background) adult male mice (8–10 weeks) and age-matched wild type (WT) C57BL/6 male mice. These animal experiments were approved by the Animal Care and Use Committee of First Affiliated Hospital of Xiamen University, China. A schematic diagram of the experimental design in vivo was shown in Fig. S1. Pgam5^−/−^ and WT mice were numbered by computer and randomly divided into TBI and sham groups. About 60 mice were used in each group.

The CCI device was used to establish a TBI mice model in vivo [20]. Briefly, a 0.5 mm diameter hole was drilled in the right parietal cortex, 2 mm posterior to the bregma and 2 mm lateral to the sagittal suture. The CCI device (PinPoint™ PCI3000; Hatteras Instruments Inc., Cary, North Carolina, USA) was adjusted to a common parameter (velocity: 5.0 m/s; depth: 2 mm; dwell time: 100 ms). The sham group underwent normal surgical procedures and not CCI. After CCI treatment, the skull was sealed and the incision was sutured. The mice were placed on a heating pad until they regained consciousness and recovered gross locomotor function. After that, the mice were put back into normal feeding units and monitored.

### Neurobehavioral analysis

Neurobehavioral training and evaluation procedures were carried out according to a previous study [19]. Experimenters were unaware of the TBI or sham treatments in WT and Pgam5^−/−^ mice. The mNSS, Rotarod, and open field tests were performed to evaluate neurological deficits by double-blind experiment. Before the experiments, all Pgam5^−/−^ and WT mice underwent behavioral experiments and were behaved as expected for a healthy animal.

### Analysis of cerebral edema

As described in previous studies [19, 21], brain water content was measured in 3-mm coronal sections of the ipsilateral cortex, centered upon the impact site. Tissues were immediately weighed (wet weight), then dehydrated at 100 °C for 24 h to obtain the dry weight. The water content of the brain was calculated using the following formula: [(wet weight–dry weight)/wet weight] × 100.

### Magnetic resonance Imaging (MRI) scanning

At 48 h post-TBI, the mice were anesthetized by the isoflurane and T2-weighted images were acquired using a 9.4 T small animal PharmaScan 94/20 MRI scanner (Bruker BioSpin, Ettlingen, Germany) according to the following parameters: repetition time/echo time (TR/TE) = 3000/50 ms; field of view 20 × 20 mm, matrix 256 × 256 mm; coronal slice thickness = 0.5 mm; number of slices = 40; scan time = 19 min. The lesion was acquired based on the high-signal area of T2-weighted images.

### Histopathological analysis

As described in the previous study [16], brain tissues were obtained after transcardial perfusion, and TUNEL/NeuN staining and Nissl staining were conducted. The Golgi-Cox staining was performed using the FD Rapid GolgiStain Kit following the manufacturer’s instructions (FD NeuroTechnologies, Columbia, MD, USA). 6 random high-power fields were chosen and images were collected using a positive fluorescence microscope (Olympus, Osaka, Japan).

### RNA sequencing (RNA-seq) and data analysis

At 48 h post-TBI, the samples of cortical tissue proximal to or located in the injury site were sent to LC Sciences (Hangzhou, China) for RNA-seq library preparation. After cluster generation, Transcriptome sequencing was carried out on an Illumina Novaseq™ 6000 platform that generated raw reads. The data that support the findings of this study have been deposited in the CNSA (https://db.cngb.org/cnsa/) of CNGBdb with accession number CNP0000970. After removing adaptor sequences, ambiguous ‘N’ nucleotides (with the ratio of ‘N’ greater than 5%) and low-quality sequences (with a quality score less than 10), the remaining clean reads were assembled using Trinity software as described for de novo transcriptome assembly with a reference genome. The mapped clean-read number was normalized to RPKM (reads per kilo of per million mapped reads). We used the edgeR package to determine the StringTie genes. Threshold of significant difference was |log2foldchange | ≥1, *p* < 0.05. The Gene Ontology (GO) enrichment analysis, Kyoto Encyclopedia of Genes and Genomes (KEGG) pathway, Heatmap analysis, and VolcanoPlot analysis were conducted at https://www.lc-bio.cn/ (LC Sciences, Hangzhou, China).

### Il-1β analysis

Commercial enzyme-linked immunosorbent assay (ELISA) kits (Beyotime, Shanghai, China) were used for measuring Il-1β levels in cortical tissue or supernatant as per the manufacturer’s instructions.

### Immunofluorescence analysis

Paraffin section of brain tissue was obtained after transcardial perfusion and primary cortical microglia was fixed by 4% paraformaldehyde after LPS + ATP treatment, then followed by overnight incubation with primary antibodies: primary anti-Cd11b antibodies (ab184308, Abcam, Cambridge, UK), anti-Gfap (#80788, CST, MA, USA), anti-Nlrp3 antibodies (19771-1-AP, Proteintech, Wuhan, China), anti-caspase1 antibodies (22915-1-AP, Proteintech), anti-caspase6 antibodies (ab185645, Abcam), anti-Il-1β (#12242, CST), and anti-α-tubulin (#3873, CST), and then incubated with secondary antibodies. Cell nuclei were stained with DAPI, 6 random high-power fields were chosen and images were obtained using a fluorescence microscope (Leica, Oskar-Barnack, Germany).

### Co-immunoprecipitation (CoIP)

Samples of cortical tissue or primary cortical microglia were lysed using a Pierce IP lysis buffer (Thermo Scientific) with a proteinase inhibitor cocktail (Roche, Basel, Switzerland). After centrifugation, suspensions were collected. CoIP was performed using a Dynabeads™ Co-Immunoprecipitation Kit (Thermo Scientific), anti-Nlrp3 (ab263899, Abcam), and anti-Asc (#67824, CST) antibodies were coupled to Dynabeads, and IgG served as the negative control. The total proteins were mixed with antibody-coupled Dynabeads and incubated overnight at 4 °C. Adsorbed Dynabeads were washed with wash buffer, and bound proteins were eluted with 20 μL eluent, mixed with 20 μL 2 × Laemmli buffer, and then boiled for 5 min. Finally, samples were loaded onto 4%–20% BeyoGel™ Plus PAGE (Beyotime) for electrophoresis.

### Western blot analysis

The total protein from cortical tissue or primary cortical microglia was lysed by the lysis buffer (Sigma-Aldrich), and a BCA protein kit (Thermo Scientific) was used to quantify protein concentration. Following SDS-PAGE electrophoresis and western transfer, a PVDF membrane (Millipore, Billerica, MA, USA) was blocked with 5% bovine serum albumin (Sigma-Aldrich) and incubated at 4 °C for overnight with the primary antibodies: Pgam5 (ab131552, Abcam), Nlrp3 (ab263899, Abcam), caspase1 (ab179515, Abcam), caspase1 (p20) (AG-20B-0042-C100, AdipoGen), Asc, Gsdmd (Gasdermin-D) (ab219800, Abcam), caspase8 (#4790, CST), Cleaved caspase8 (#8592, CST), Il-1β (#12242, CST), Cleaved Il-1β (#63124, CST), and Gapdh (#2118, CST).

For chemical cross-linking, the monomer and oligomer of Asc (#67824, CST) and Pgam5 (ab131552, Abcam) were detected as described by Fernandes-Alnemri and his colleagues [22]. Then crude pellet was resuspended in CHAPS buffer and was chemically crosslinked with 4 mM non-cleavable disuccinimidyl suberate (DSS) cross-linker for 30 min. The samples obtained were used for Western blot analysis.

### qRT-PCR analysis

The total RNA was isolated using Trizol reagent (Invitrogen, Waltham, MA, USA). A HiFi-MMLV cDNA First-Strand Synthesis Kit (CW Bio, Beijing, China) was used for reverse transcription. GoTaq qPCR Master Mix (Promega, WI, USA) was used for qRT-PCR analysis on the CFX96TM real-time system (Bio-Rad, CA, USA). The expression of the genes of interest was normalized to the levels of Gapdh. Primer sequences are listed in Table S1:

### Primary cortical microglia culture

Pure neonatal microglia cultures were obtained from the cortices of neonatal Pgam5^−/−^ or WT mice as previously described in detail [23]. Briefly, cortices were incubated in trypsin/EDTA solution for 15 min at 37 °C, and then the culture medium (DMEM with the addition of 10% fetal calf serum, 1% penicillin/streptomycin, and 2 mM L-glutamine) was added to stop the reaction. After dissociated the cortices, the suspension was centrifuged at 1000 rpm for 2 min. Cells were resuspended in culture medium and grown in 75 ml flasks at 37 °C with 5% CO_2_, while changed the culture medium every 3 days. After 2 weeks in culture, the cells were isolated by gently shaking of the flask (250 rpm) at 37 °C for 1 h to detach microglia, and collected the medium and immediately centrifuged for 2 min at 1200 rpm, and the obtained pure microglia pellet was re-suspended in fresh culture medium and seeded into subcultures. About 95% of these cells were positive for Cd11b, a marker for microglia cell types. Experiments were started 24 h after cultivation.

### Transfection in primary cells

Pgam5-shRNA Lentiviral Particles (sc-152184-V; Santa, CA, USA), Rip3-shRNA Lentiviral Particle (sc-61483-V; Santa), and caspase8-shRNA Lentiviral Particles (sc-37226-V; Santa) were used for transfection following the manufacturer’s instructions in primary cortical neurons or microglia. At 48 h after infection, transfection efficiency was assessed using qRT-PCR analysis (Fig. S2).

### Microglial viability and activation

10 ng/mL LPS (Sigma-Aldrich) + 5 mM ATP (Sigma-Aldrich) were used to in vitro [16]. After LPS treatment for 4 h and ATP for an additional 10, 20, 30, or 45 min, cells and medium supernatant were collected for subsequent detections. Cell Counting Kit-8 (CCK8; Dojindo Laboratories, Tokyo, Japan) was used to determine cell viability. Griess reaction (Solarbio, Beijing, China) to measure the NO production in microglia.

### Asc speck staining

Cells were blocked with 5% goat serum after fixation and permeabilization, then incubated with anti-Asc antibody (#67824, CST) at 4 °C overnight and with secondary antibody (Alexa Fluor 594-conjugated, Abcam) at 37 °C for 2 h. After stained cytoblast with DAPI, images were collected using an inverted fluorescence microscope (Olympus).

### Statistical analysis

All the data were presented as the mean ± SEM and were analyzed using SPSS statistical software (version 22.0, IBM, Armonk, NY, USA) and GraphPad Prism 5 software (San Diego, CA, USA). Sample size and animal numbers were determined based on previous studies. Before the analysis, all data were checked for normality and homogeneity of variances. Two-tailed independent-sample *t*-test and one-way and two-way analysis of variance (ANOVA) followed by Tukey’s post hoc multiple comparison tests were used. The variance is similar between the groups that are statistically compared. A *p* value < 0.05 was considered to be statistically significant.

## Results

### Pgam5 depletion relieves neurological deficits and nerve injury after TBI

**Fig. 1:**
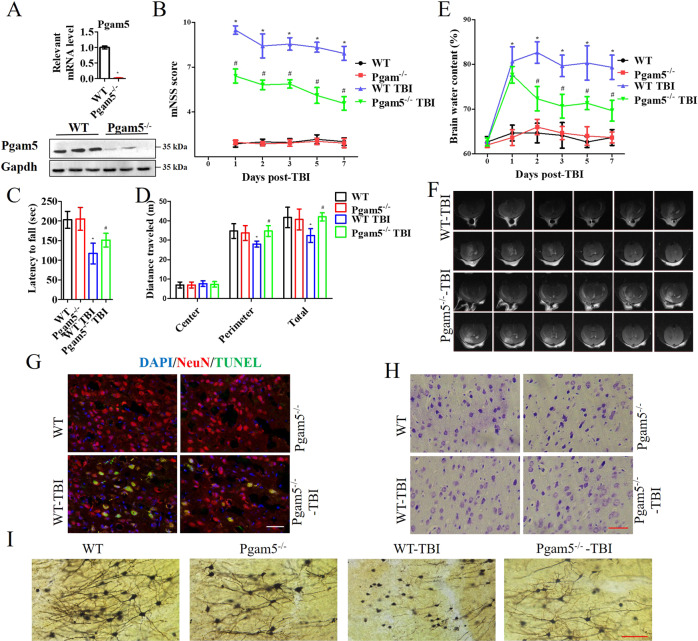
Pgam5 deficiency improves neurological deficits, neuron damage, and neuronal neurites degeneration after TBI. **A** Western blot and RT-PCR results showed that Pgam5 expression was abolished in Pgam5^−/−^ mice. Neurological performances were assessed by (**B**) mNSS, (**C**) Rotarod test, and (**D**) open field test, *n* = 8. **E** At 1, 2, 3, 5, and 7 days after TBI in WT and Pgam5^−/−^ mice, the brain water content was detected. **F** At 2 days after TBI, the T2-weighted MRI images showed histological impairments. **G** Cortical injuries to coronal sections were assessed using TUNEL staining 48 h post-TBI. Nuclei were stained with DAPI and neurons were stained with NeuN. **H** Nissl staining was performed to determine the morphological changes and damage of cortex neurons after TBI. **I** Representative pictures from cortical sections show prominent Golgi-Cox staining of degenerating neurites 2 days post-TBI. The images shown are representative of typical images from 6 mice in each group. E~I, *n* = 6. Data shown are means ± SEM, *p < 0.05 compared to the WT group and ^#^*p* < 0.05 com*p*ared to WT TBI group by two-way ANOVA followed by Tukey’s post hoc multiple comparison test.

In Pgam5^−/−^ mice, the mRNA and protein levels of Pgam5 were near negligible in comparison to WT mice (Fig. 1A). Before the tests, no differences in mNSS, Rotarod, or open field scores were observed between the groups. 7 days after TBI, Pgam5^−/−^ mice showed lower mNSS scores compared to WT mice (Fig. 1B). In the Rotarod test (Fig. 1C), WT-TBI mice were easy to fall off the stick, but the time of latency to fall of Pgam5^−/−^-TBI mice was better compared to the WT-TBI mice. In the open field test (Fig. 1D), WT-TBI mice significantly moved less in the perimeter region resulting in less total distance, and Pgam5^−/−^-TBI mice showed a significant increase in the activity of the perimeter region and the total distance.

TBI induced significant brain tissue edema, the score was maximal at 2 d and gradually decreased, but still with significant difference compared to the sham groups (Fig. 1E). Pgam5 deficiency significantly alleviated this phenomenon compared with WT-TBI mice from day 2 (Fig. 1E). T2-weighted MRI images were also used to evaluate the effect of Pgam5 deletion at 48 h post-TBI (Fig. 1F) and Pgam5 deficiency alleviated histological impairments. The TUNEL/NeuN positive cells were significantly enhanced in WT-TBI mice, but that significantly reduced in Pgam5^−/−^-TBI mice (Fig. 1G). After TBI, the damaged neuron cells had increased, presenting extensive degenerative changes, including less Nissl body staining. Inversely, severe nerve injury was seen to be significantly reduced in Pgam5^−/−^-TBI mice (Fig. 1H). Pgam5 depleted blocked the reduction in the arborization of neurites after TBI (Fig. 1I).

### Pgam5 facilitates Il-1β activation and microglia activation after TBI

**Fig. 2:**
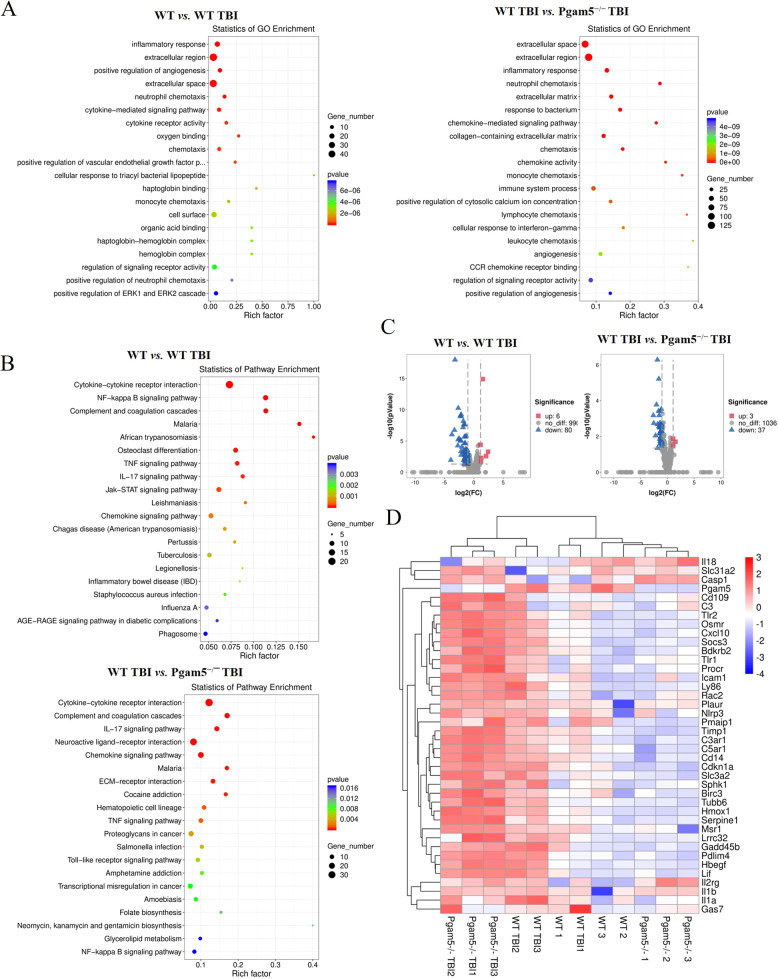
RNA-seq analysis of cortical tissue. Total 55,450 genes of cortical tissues were detected by LC Sciences for RNA-seq analysis. **A** GO enrichment analysis was carried out to classify the biological function of DEGs. Each box shows the GO term number, the p-value, and GO term. **B** KEGG pathway enrichment analysis for DEGs. The top 20 KEGG pathways are shown. The box color indicates the level of statistical significance. The dot size means the gene number. **C** A 1076 genes associated with inflammatory response were isolated and analyzed by VolcanoPlot. **D** Heatmap analysis of inflammation-related DEGs between WT group, WT TBI group, Pgam5^−/−^ group, and Pgam5^−/−^ TBI group. Only the top 40 genes were included in the DEGs heatmap.

To classify the biological function of the differentially expressed genes (DEGs), a GO enrichment analysis was carried out. As shown in Fig. 2A, the enriched DEGs were mainly associated with the inflammatory response (GO:0006954), and the extracellular region (GO:0005576). The KEGG enrichment analysis showed that the cytokine-cytokine receptor interaction (ko04060), NF-kappa B signaling pathway (ko04064), Tnf signaling pathway (ko04668), and Il-17 signaling pathway (ko04657) were enriched (Fig. 2B), indicating that Pgam5 in regulating inflammatory response through regulating these genes’ expression patterns. To assess the role of Pgam5 on neuroinflammation, 1,076 genes associated with inflammatory response were isolated and analyzed. 3 genes were up-regulated and 37 genes were down-regulated when WT-Ctl were compared to WT-TBI; 6 genes were up-regulated and 80 genes were down-regulated when WT-TBI were compared to Pgam5^−/−^-TBI (Fig. 2C). Heatmap analysis showed TBI decreased Dsc2, Traf1, and Fsd1l mRNA levels but induced Il-1β, Hmox1, Cxcl10, and Nlrp3. expression (Fig. 2D). Interestingly, Pgam5 deletions did not mediate Il-1β, Hmox1, Cxcl10, and Nlrp3. mRNA levels (Fig. 2D), that might regulate inflammatory responses by affecting the post-translational modification and protein expression of related genes, including Nlrp3, caspase1, Gsdmd, and Il-1β.

**Fig. 3:**
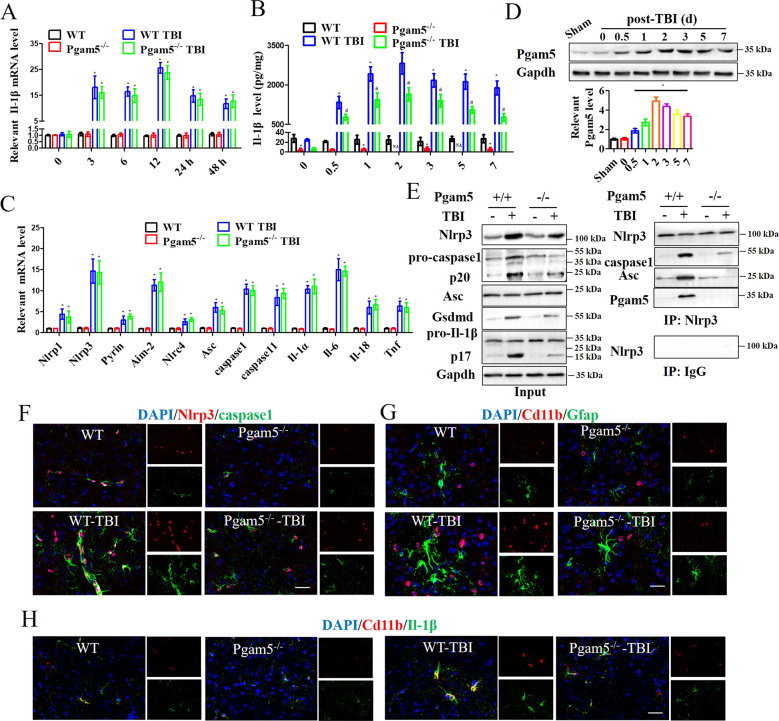
Pgam5 depletion relieves TBI-induced Il-1β through Nlrp3 inflammasome activation and Pgam5 is involved in TBI-induced microglial activation in vivo. The Il-1β level was monitored after TBI in WT and Pgam5^−/−^ mice. **A** qRT-PCR analysis of Il-1β mRNA level and (**B**) ELISA analysis of Il-1β level. **C** Nlrp1, Nlrp3, Pyrin, Aim-2, Nlrc4, Asc, caspase1, caspase11, Il-1α, Il-6, Il-18, and Tnf mRNA in Pgam5^−/−^-TBI mice were measured at 24 h after TBI. (**A**, **B** and **C**) *p < 0.001 compared to the WT group and #*p* < 0.001 compared to the WT TBI group by two-way ANOVA followed by Tukey’s post hoc multiple comparison test. **D** Western blot analysis of Pgam5 expression during 7 days after TBI in the cortex. The lysate of the cortex was analyzed using Western blot assay to detect the Nlrp3/caspase1/Il-1β pathway at 48 h after TBI. *p < 0.001 compared to the sham group by one-way ANOVA followed by Tukey’s post hoc multiple comparison test. **E** Results of immunoblotting with antibodies to the indicated proteins and immunoprecipitated with anti-Nlrp3. At 48 h after TBI in WT and Pgam5^−/−^ mice, cortical sections were analyzed by immunofluorescence for the expression of (**F**) Nlrp3/ caspase1, (**G**) Cd11b/Gfap, and (**H**) Cd11b/Il-1β. Nuclei were stained with DAPI. Scale bar: 20 μm. Data shown are means ± SEM, *n* = 3.

As shown in Fig. 3A, pyroptosis and inflammation molecules were still high mRNA levels Pgam5^−/−^-TBI mice after TBI. Il-1β mRNA level in cortex significantly increased within 48 h after TBI, mRNA levels peaked at 12 h, and then it went down (Fig. 3A). However, the mRNA level was still 23.7 ± 4.6-fold relative change at 12 h in Pgam5^−/−^-TBI mice (Fig. 3A). Inversely, Il-1β levels peaked 2427 ± 434 pg/mg at 48 h and then gradually declined after TBI, while this was significant in the Pgam5^−/−^-TBI group (Fig. 3B). However, Pgam5 deficiency did not affect the pyorptosis-related gene expression levels (Fig. 3C), which was consistent with the result of RNA-seq. Pgam5 level was at the same tendency with Il-1β level and appeared to peak at 2 days after TBI (Fig. 3D). The data suggested that Il-1β might be linked to Pgam5. TBI triggered the recruitment of Pgam5 to Nlrp3 complex, and Pgam5 deficiency suppressed the assembly of Nlrp3 inflammasome, including Nlrp3 interacting with caspase1 and Asc, and Il-1β was regulated by Nlrp3-mediated pyroptosis (Fig. 3E). Furthermore, a high level of Cd11b appeared around the damaged cortical tissue after TBI, and TBI increased the interaction with Nlrp3 and caspase1 (Fig. 3F), and induced Cd11b and Gfap positive cells, indicating the activation of microglia and astrocytes (Fig. 3G). In Pgam5^−/−^-TBI mice, Cd11b/Il-1β positive cells were significantly less and the interaction with Nlrp3 and caspase1 was blocked compared with the WT-TBI group (Fig. 3F–H). The data suggest that Pgam5 facilitates microglia activation-mediated Il-1β and acute inflammation, which may be involved in the pathology of nerve damage.

### Pgam5 is required for microglia-induced Il-1β secretion via activating Asc oligomerization

**Fig. 4:**
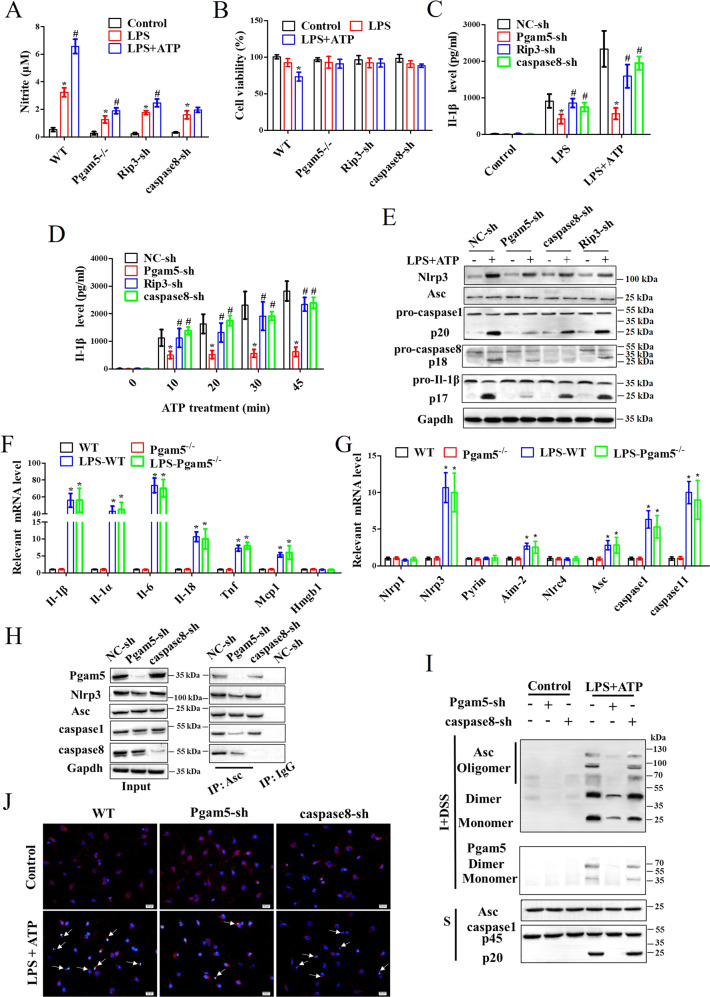
Pgam5 is required for microglia-induced Il-1β secretion and Asc oligomerization. NO production was detected by the (**A**) Griess reaction and cell viability was measured by (B) CCK8 assay. (**A**) ^*^p < 0.001 compared to the control group and ^#^*p* < 0.001 compared to the LPS group, by two-way ANOVA followed by Tukey’s post hoc multiple comparison test. **C** Primary microglia were primed with LPS for 4 h prior to stimulation with ATP for 30 min, and Il-1 β secretion was determined by ELISA. **D** Dynamically monitor Il-1β level in LPS-primed microglia treated with ATP for the indicated amount of time. **C**, **D** ^*^p < 0.001 compared to the NC-sh group and ^#^*p* < 0.001 compared to the Pgam5^-^sh group, by two-way ANOVA followed by Tukey’s post hoc multiple comparison test. (**E**) Whole-cell extracts were subjected to Western blot analysis. Microglia were stimulated with LPS + ATP and the mRNA of (**F**) cytokineand (**G**) pyroptosis-related genes were determined by qRT-PCR. ^*^p < 0.001 compared to the WT group by one-way ANOVA followed by Tukey’s post hoc multiple comparison test. Data shown are mean ± SEM (*n* = 4). Pgam5-shRNA and caspase8-shRNA microglia were stimulated with LPS + ATP. **H** Results of immunoblotting with antibodies to the indicated proteins and immunoprecipitated with anti-Asc. **I** The detergent-insoluble fraction was subjected to chemical cross-linking, and monomers, dimers, and oligomers of Asc and Pgam5 were measured. **J** Immunofluorescence analysis of Asc specks (arrows) in microglia. After treatments, cells were fixed and stained for Asc (Red). Nuclear staining with DAPI (Blue). *n* = 4.

There was a significant decrease in nitrite in LPS-induced Pgam5^−/−^, Rip3-shRNA, and caspase8-shRNA microglia compared with WT microglia (Fig. 4A). LPS + ATP suppressed cell viability in WT microglia but not in Pgam5^−/−^, Rip3-shRNA, and caspase8-shRNA microglia (Fig. 4B). As shown in Fig. 4C, LPS + ATP significantly induced Il-1β secretion, while Il-1β secretion was significantly suppressed in Pgam5^−/−^ microglia after stimulation related to LPS + ATP-induced WT microglia but not in Rip3-shRNA and caspase8-shRNA microglia (Fig. 4C). Time-course analysis showed that LPS-induced Il-1β secretion by Pgam5^−/−^ was reduced at all time points tested, but not influenced by Rip3-shRNA and caspase8-shRNA (Fig. 4D). LPS + ATP stimulation activated the Nlrp3 inflammasome in primary microglia, which manifested as caspase1 p20 and Il-1β p17 secretion in an Nlrp3/Asc-dependent manner. Reduced levels of activated caspase1 (p20) and Il-1β p17 were measured in LPS + ATP-induced Pgam5^−/−^ microglia (Fig. 4E). Indeed, caspase8 was activated in Pgam5^−/−^ and WT microglia, and Rip3 or caspase8 downregulation does little to regulate caspase-1 p20 and IL-1β p17 secretion in LPS + ATP-induced microglia (Fig. 4E). Pgam5 downregulation reduced mature Il-1β secretion, but not mRNA levels of Il-1β and other inflammatory cytokines (Fig. 4F). LPS-induced high levels of pyroptosis-related genes were still in Pgam5^−/−^ microglia (Fig. 4G).

CoIP results showed that interaction with Asc and Nlrp3, caspase1, and caspase8 were significantly decreased in Pgam5^−/−^ microglia compared with WT microglia, but caspase8-shRNA did not affect this phenomenon (Fig. 4H). In WT microglia, LPS + ATP-induced Asc oligomerization was measured concomitant with the appearance of caspase1 p20 (Fig. 4I). ASC translocation to an insoluble compartment and Asc oligomerization were significantly reduced in Pgam5^−/−^ microglia, but weak variation was seen in caspase8-shRNA microglia (Fig. 4I). Furthermore, LPS + ATP drove Pgam5 translocation to the insoluble compartment and Pgam5 oligomerization (Fig. 4I). After stimulation of LPS or LPS + ATP in microglia, large intracellular Asc specks were rapidly formed in the cytosol. Asc specks were reduced in Pgam5^−/−^ microglia, but weak variation was seen in caspase8-shRNA microglia (Fig. 4J). Although some reports have shown that caspase8 participates in the processing of pro-Il-1β under certain conditions [24, 25]. the data suggest that Pgam5 facilitates the Asc-dependent pro-Il-1β processing via caspase1-associated inflammasome that is independent of Rip3/caspase8 pathway in LPS + ATP-stimulated microglia.

## Discussion

Microglia are the primary mediators of the innate immune response in CNS, and the proinflammatory cytokines released by microglia, such as Il-1β and Tnf-α, are important assessment indices of microglia polarization and pro-inflammatory after TBI [24]. Indeed, inhibiting microglia activation-mediated inflammation is suggested to improve the neurological outcomes post-TBI [25]. In this study, we have investigated the molecular mechanism of Pgam5 after TBI. Pgam5 deletion reduced TBI-induced Nlrp3/caspase1/Il-1β pathway activation, microglial activation, and neurological deficits in vivo. Pgam5 facilitated Asc polymerization and Il-1β secretion in microglia, and Pgam5 downregulation alleviated LPS + ATP-induced microglia activation and Il-1β via Asc/caspase1-mediated pyroptosis.

Neuroinflammation is one of the leading causes of secondary brain injury, and its reduction is regarded as an excellent choice to improve the prognosis of TBI [26, 27]. In clinical practice, Helmy et al. have demonstrated that the injection of recombinant IL-1R antibody reduces IL-1-mediated brain injury post-TBI by alleviating neuroinflammation [28]. Moriwaki et al. have verified that Pgam5 promotes inflammasome activation including Il-1β secretion in BMDMs [17]. Our results showed Pgam5 deficiency reduced Nlrp3 inflammasome assembly, caspase1 activation, and Il-1β activation by inhibiting Asc polymerization in microglia, suggesting that Pgam5 also facilitated pyroptosis via Asc/caspase1 pathway.

As opposed to caspase8-mediated Il-1β secretion and inflammation, Pgam5 has also been seen to be involved in LPS-induced Il-1β secretion in caspase8-deficient BMDCs [29]. Moreover, caspase8 deficiency enhances LPS-induced Nlrp3 inflammasome assembly and function, and Pgam5 contributes to Rip3-mediated induction of necrosis and Nlrp3 inflammasome activation in dendritic cells [29]. However, Moriwaki et al. have shown that primary mouse embryonic fibroblasts with Pgam5 deficiency responds normally to multiple inducers of apoptosis and necroptosis, and vesicular stomatosis virus-induced Il-1β secretion is reduced in Pgam5^−/−^ BMDMs but not in BMDMs with Rip3 deficiency, suggesting that Pgam5-induced inflammation activation is independent of Rip3 [17]. Caspase1 inhibition prevents glial inflammasome activation and pyroptosis in cortical astrocytes and multiple sclerosis models [30, 31]. VX-765 (50 μM) suppresses caspase1 activation and Il-1β in astrocytes treated with unconjugated bilirubin but not pro-caspase1 level [30]. Our study demonstrated Pgam5 does not regulate the gene transcription of cytokines but rather participated in the processing of pro-Il-1β, and Pgam5 participated in Asc/caspase1-mediated microglia activation and pyroptosis. Phillips et al. have identified complex crosstalk and redundancy between caspase1 and caspase8 in Il-1β processing in bone disease [32]. Kang et al. have demonstrated that caspase8 enzymatic activity is not required for dsRNA-induced Asc polymerization [33]. The results suggest that Pgam5 may be an upstream regulatory molecule of microglia in the Asc/caspase1/Il-1β pathway under certain circumstances, such as infected BMDMs and nerve injury, which is independent of caspase8/Rip3 signal but via Asc polymerization.

Generally, Pgam5 is identified as a moderator dephosphorylating and activating Drp1, Bcl-xL, Fundc1, etc., which involves mitochondria fission and mitophagy to maintain normal function and state of mitochondria [9, 34]. In this present work, Pgam5 depletion alleviated LPS-induced microglia activation and Il-1β via Asc/caspase1-mediated pyroptosis. Numerous studies have shown that Il-1β influences the survival of a variety of cells [35, 36], including cortical neurons and hippocampal neurons [31, 36]. Cai et al. have demonstrated that intracerebral injection of Il-1β leads to acute white matter and neuronal injury in neonatal rats [37], and Fan et al. have shown that Il-1β inhibition improves the associated neurological dysfunctions in juvenile rats [38].

## Conclusion

In this study, we have discussed the mechanism of Pgam5 in nerve injury after TBI. Pgam5 facilitates microglial inflammasome activation to Asc/caspase1-generated Il-1β-mediated neuroinflammation after TBI, and TBI-induced neuroinflammation may be partly dependent on the interaction between Pgam5 and Nlrp3 inflammasome and Asc oligomerization. Thus, Pgam5 may be a potential therapeutic target to improve neuroinflammation and nerve injury after TBI.

## Supplementary information

Table S1

Figure S

Figure S1

Figure S2

## Data Availability

The datasets and computer code produced in this study are available in the following databases, RNA-Seq data: Gene Expression, Omnibus CNP0000970 (http://db.cngb.org/cnsa/).
