# Supplementary material for: Downregulation of phosphoglycerate mutase 5 improves microglial inflammasome activation after traumatic brain injury
Source: Cell Death Discov. 2021 Oct 12;7:290. doi: 10.1038/s41420-021-00686-8 (PMC8511105; doi:10.1038/s41420-021-00686-8)
Supplement: Supplementary file 1 — Table S1 [file 41420_2021_686_MOESM1_ESM.docx]

Table S1. Primers for RT-qPCR

|  | Primer | Sequence（5'-3'） |
| --- | --- | --- |
| Il-1α | Forward primer | AAGACAAGCCTGTGTTGCTGAAGG |
|  | Reverse primer | TCCCAGAAGAAAATGAGGTCGGTC |
| Il-1β | Forward primer | GCTTCAGGCAGGCAGTATC |
|  | Reverse primer | AGGATGGGCTCTTCTTCAAAG |
| Il-6 | Forward primer | ACCGCTATGAAGTTCCTCTC |
|  | Reverse primer | CTCTGTGAAGTCTCCTCTCC |
| Il-18 | Forward primer | ACCAAGTTCTCTTCGTTGAC |
|  | Reverse primer | TCACAGCCAGTCCTCTTAC |
| Nlrp1 | Forward primer | GGTGTGCTGGTTGGTCTGC |
|  | Reverse primer | GTGCTGTGGTGGTCTGTGAG |
| Nlrp3 | Forward primer | GCTCCAACCATTCTCTGACC |
|  | Reverse primer | AAGTAAGGCCGGAATTCACC |
| Nlrc4 | Forward primer | GTCAAGTGTTATCCAAGTTA |
|  | Reverse primer | CGCTAATATCATAGTCATCAA |
| Aim2 | Forward primer | ATAGGAGGAACAACAACAT |
|  | Reverse primer | GCCATCTTCTGCTACATA |
| Asc | Forward primer | AGGAGTGGAGGGGAAAGC |
|  | Reverse primer | AGAAGACGCAGGAAGATGG |
| caspase-1 | Forward primer | AGGAATTCTGGAGCTTCAATCAG |
|  | Reverse primer | TGGAAATGTGCCATCTTCTTT |
| caspase-11 | Forward primer | GCTCTTACTTCATCACTA |
|  | Reverse primer | AATATCTCGTCAAGGTTG |
| Tnf | Forward primer | GGTTCTGTCCCTTTCACTCAC |
|  | Reverse primer | TGCCTCTTCTGCCAGTTCC |
| Pyrin | Forward primer | TTCATTGGGAGCACCTTGGG |
|  | Reverse primer | GCAGCTGCTTGGTCCAAATC |
| Gapdh | Forward primer | AACTTTGGCATTGTGGAAGG |
|  | Reverse primer | GGATGCAGGGATGATGTTCT |
