# Supplementary material for: Downregulation of phosphoglycerate mutase 5 improves microglial inflammasome activation after traumatic brain injury
Source: Cell Death Discov. 2021 Oct 12;7:290. doi: 10.1038/s41420-021-00686-8 (PMC8511105; doi:10.1038/s41420-021-00686-8)
Supplement: Supplementary file 2 — Figure S [file 41420_2021_686_MOESM2_ESM.docx]

**Figure Legends:**

**Figure S1. Schematic diagram of the experimental design in vivo**. Before the CCI model, all mice of Pgam5^−/−^ and WT were tested to be normal by behavioral tests, and then randomly assigned to TBI and sham operation. After TBI, some mice were used for MNSS, Brain Water Content, ELISA, and WB experiments (at 0, 1, 2, 3, 5, and 7 days after TBI), some mice were used for qRT-PCR analysis (at 0, 1, and 2d after TBI), and other mice were used to perform open-field test, Rotarod test, MRI, TUNEL/Nissl/Golgi-Cox staining, RNA-seq, WB, IF experiments (at 48 h after TBI).

**Figure S2.** qRT-PCR analysis of mRNA expression level in primary microglia after transfection. Primary microglia was infected with Pgam5-shRNA Lentiviral Particles, Rip3-shRNA Lentiviral Particle, caspase8-shRNA Lentiviral Particles, and the Control shRNA (sc-108080) according to the manufacturer’s protocol (Santa Cruz Biotechnology, Inc.). Pgam5 (A, Pgam5-shRNA and Control shRNA were used), Rip3 (B, Rip3-shRNA and Control shRNA were used), and caspase8 (C, caspase8-shRNA and Control shRNA were used) mRNA level were measured. Data were represented as means ± SEM, one representative experiment of three was shown. **p* < 0.05, vs the control group.
