# Supplementary figures and images for: Downregulation of phosphoglycerate mutase 5 improves microglial inflammasome activation after traumatic brain injury
Source: Cell Death Discov. 2021 Oct 12;7:290. doi: 10.1038/s41420-021-00686-8 (PMC8511105; doi:10.1038/s41420-021-00686-8)

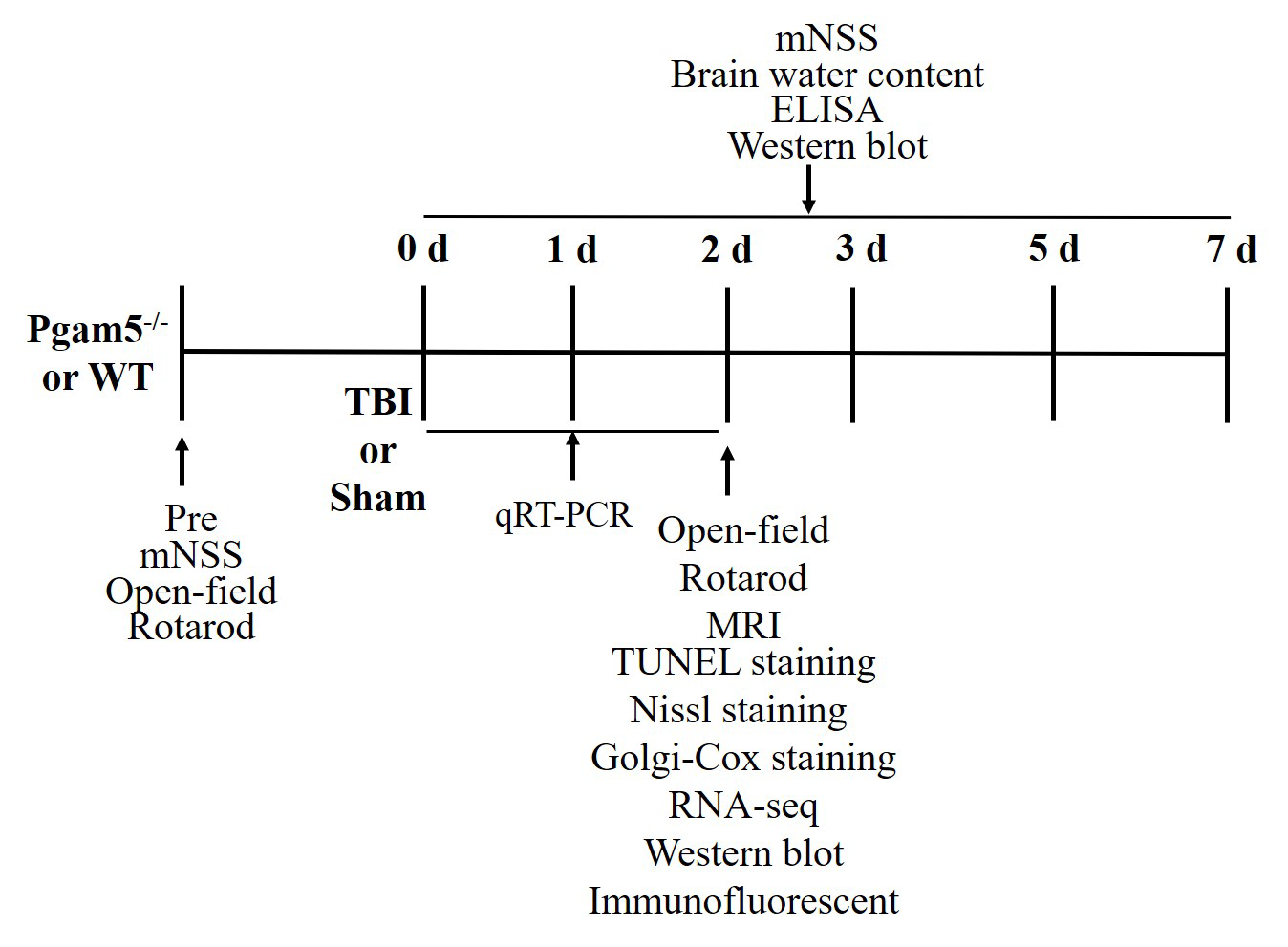

Supplement: Supplementary file 3 — Figure S1 [file 41420_2021_686_MOESM3_ESM.tif]

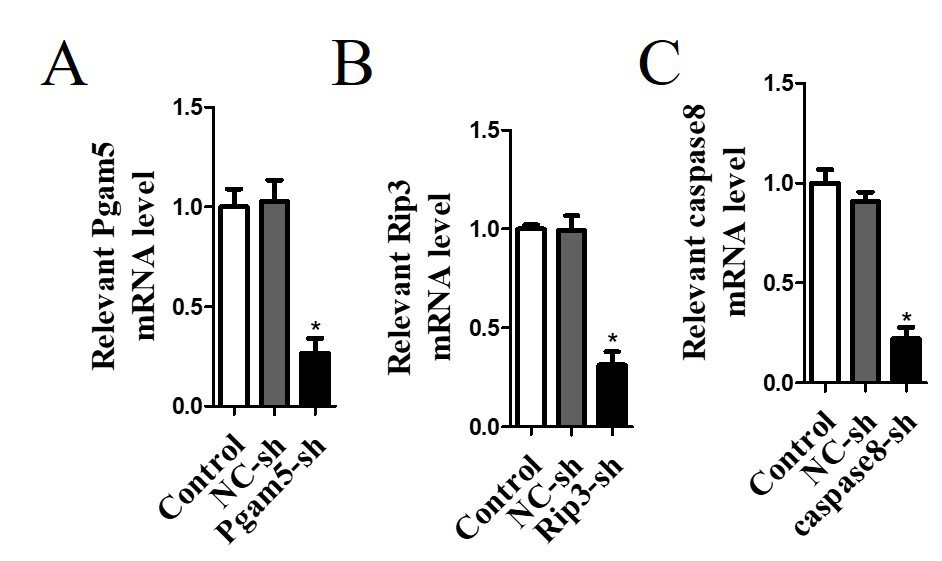

Supplement: Supplementary file 4 — Figure S2 [file 41420_2021_686_MOESM4_ESM.tif]
